# Translation and validation of the international consultation on incontinence questionnaire-vaginal symptoms: the simplified Chinese version

**DOI:** 10.1007/s00192-022-05329-9

**Published:** 2022-08-24

**Authors:** Yufeng Liu, Yingyang Li, Tao Zhu, Tiantian Jia, Kexin Jiang, Enshe Jiang

**Affiliations:** 1 Institute of Nursing and Health, Henan University, Kaifeng, 475004 Henan China; 2 Luoyang Central Hospital affiliated to Zhengzhou University, Luoyang, 471000 Henan China; 3 Department of Geriatrics, Kaifeng Traditional Chinese Medicine Hospital, Kaifeng, 475001 China; 4 Department of Nursing, Henan Provincial Chest Hospital, Zhengzhou, 450008 Henan China

**Keywords:** Chinese version, Incontinence questionnaire, Vaginal symptoms, Validation

## Abstract

**Introduction and hypothesis:**

International Consultation on Incontinence Questionnaire-Vaginal Symptoms (ICIQ-VS) is a simple and effective questionnaire for evaluating vaginal symptoms, sexual problems and the quality of life (QOL) in patients. This study was aimed at validating the simplified Chinese version of the ICIQ-VS.

**Methods:**

A total of 120 women with pelvic organ prolapse (POP) stage <2, 124 with stage ≥ 2, and 51 patients who underwent POP surgery (POP stage ≥2) were included. Cronbach’s alpha coefficient and intraclass correlation coefficient (ICC) were used for reliability analysis. We used the content validity index, Kruskal–Wallis *H* test, and Mann–Whitney *U* test to study validity. Paired sample *t* test, Wilcoxon signed-rank test, effect size and standardized response mean were used to assess sensitivity.

**Results:**

The Cronbach’s alpha coefficients of the vaginal symptoms score (VSS) and sexual matters score (SMS) were 0.787 and 0.861 respectively. The test–retest reliabilities of the VSS, SMS, and QOL score were 0.830, 0.894, and 0.948 respectively. The test–retest reliability was from good to excellent (ICC 0.669–0.948). The item-level content validity index was 0.60 to 1.00. The scale-level content validity index/universal agreement was 0.95, and the scale-level content validity index/average was 0.96. Significant score differences existed between the symptomatic and asymptomatic groups (*p* < 0.001). Criterion validity was significant (*p* < 0.001). VSS and QOL score had high sensitivity (*p* < 0.001, effect size and standardized response mean >0.8).

**Conclusions:**

The simplified Chinese version of the ICIQ-VS can objectively and reliably access vaginal symptoms, sexual matters, and QOL in Chinese women.

## Introduction

Pelvic floor dysfunction (PFD) is a condition with clinical symptoms of pelvic organ dysfunction or abnormal position due to weak pelvic support [1]. Pelvic organ prolapse (POP) and urinary incontinence (UI) are the main symptoms of PFD and often occur in combination.

Currently, the incidence of PFD is increasing year by year. About 23–49% of women worldwide suffer from different types of PFD [2]. In recent years, the incidence of PFD in the United States has been as high as 25%, and the number of women with PFD is predicted to increase by 30.1 million between 2010 and 2050, with 15.8% of those women suffering from POP [3–5]. In China, the incidence of PFD in women is about 30%, and the incidence of symptomatic POP is about 9.6%. However, there is a general lack of awareness of the disease [6–8]. Pelvic floor dysfunction often occurs with some vaginal problems, which affect the sexual matters and the quality of life (QOL) of patients [9]. Most patients are ashamed to express their privacy issues and suffer for a long time. Only a few patients seek help from medical institutions, so the actual situation of the symptoms of patients is largely underestimated. As a simple and effective survey tool, a questionnaire can help medical personnel to evaluate the subjective symptoms and therapeutic effects of patients.

Advocated by the Third International Consultation on Incontinence (ICI), Price et al. [10] developed a questionnaire to assess the impact of vaginal symptoms, sexual matters and QOL related to PFD—the International Consultation on Incontinence Questionnaire-Vaginal Symptoms (ICIQ-VS)—in 2006. The questionnaire has been translated into Portuguese, German, Sinhalese, Tamil, Greek, Danish, and Turkish [11–16]. The questionnaire is recommended by the ICI. However, it has not been introduced into China until now. In this study, we aimed to translate the ICIQ-VS into simplified Chinese and verify its reliability, validity, and sensitivity.

## Materials and methods

The ICIQ-VS is a questionnaire developed by Price et al. in 2006. It was used to assess the impact of vaginal symptoms, sexual matters, and QOL related to PFD [10]. The ICIQ-VS comprises 14 items and three dimensions, including the vaginal symptoms score (VSS), the sexual matters score (SMS), and the QOL score. The ranges of VSS, SMS, and QOL score are 0–53, 0–58, and 0–10 respectively. The scores increase with the increase in the severity of symptoms. The researcher was authorized to translate this questionnaire into simplified Chinese after contacting the ICIQ group.

### Translation and cross-cultural adaptation

Brislin’s translation model for cross-cultural research was strictly followed during translation of the ICIQ-VS into simplified Chinese [17]. The ICIQ-VS was translated by two independent native Chinese speakers (a Master of Nursing who has passed the College English Test 6 and a Master of Clinical Medicine with an International English Language Testing System score of 6.5) who are proficient in English and have had no prior exposure to the ICIQ-VS. After discussion, the first draft of the simplified Chinese version was back-translated into English by another two native Chinese speakers (a doctor of English major and a senior gynecologist with overseas study experience) who are unfamiliar with the ICIQ-VS. After comparison and analysis by experts, the back-translation version was finally formed. The researcher submitted the first draft of the simplified Chinese version and the back-translated version to the questionnaire development group for review and made changes based on comments from the development group. Ten experts were selected to conduct cross-cultural adjustment through two rounds of Delphi interviews. Expert selection criteria were as follows:

1. Having the title of associate chief physician or above
2. Having more than 10 years of clinical work experience in the field of gynecology
3. Giving informed consent and voluntarily participating in the study

Then, during the pilot survey phase, 30 patients with POP were selected to fill out the questionnaire. According to the interview outline provided by the ICIQ-VS group, the researcher conducted and recorded interviews with patients. Finally, the researcher determined the simplified Chinese version of the ICIQ-VS. The full simplified Chinese version of the ICIQ-VS can be obtained from the ICIQ office or from the author.

### Study population

In this study, we recruited patients with POP and those without POP through convenience sampling at the Gynecological Outpatient Clinic of Henan Provincial People’s Hospital from March 2021 to June 2021. Women aged 18 years or older without language communication disorders and cognitive dysfunction were enrolled in the study. Pregnant women, patients with gynecological tumors, patients with active vulvovaginal disease, and patients in the acute phase of pelvic inflammatory disease were excluded. Based on the recommendations of the ICIQ-VS group and similar other ICIQ-VS studies, a sample size of 244 was selected [10, 15, 16]. A total of 124 patients with POP stage ≥2 were included in the symptomatic group; the asymptomatic group consisted of 120 patients with POP stage 0–1. At the first examination (T1), the patients were evaluated by an experienced gynecologist using the pelvic organ prolapse quantification (POP-Q) system and filled out the simplified Chinese version of the ICIQ-VS. Two weeks later (T2), the 30 patients with POP filled out the simplified Chinese version of the ICIQ-VS again to measure the test–retest reliability.

In addition, 51 patients with POP who underwent surgery were recruited for this study. They filled out the questionnaire before surgery (T3) and 3 months after surgery (T4) to evaluate the sensitivity of the questionnaire. The patients considered the requirement of the gynecologist to abstain from sexual activities within 3 months after the surgery, and they were concerned that premature sexual activities would affect surgery outcomes three months after the surgery. Therefore, the patients did not fill in questions about sexual matters.

Sociodemographic data (age, parity, number of cesarean sections, and history of menopause) were recorded. All the patients signed the informed consent form, and the study was approved by the Henan University Ethics Committee (approval no. HUSOM2021-2-26).

### Statistical analysis

#### Reliability

##### Internal consistency

Internal consistency is the degree of homogeneity between the items. Internal consistency of VSS and SMS was determined by Cronbach’s alpha coefficient. Cronbach’s alpha coefficient ≥0.70 indicates that the internal consistency reliability is acceptable.

##### Stability

Stability was determined by the intraclass correlation coefficient (ICC). Two weeks later, data from the 30 patients with POP (T1 and T2) were analyzed. Intraclass correlation coefficient <0.40 indicates a poor test–retest reliability; 0.40 ≤ ICC ≤ 0.75 means that the test–retest reliability is medium; ICC >0.75 indicates a good test–retest reliability.

#### Validity

##### Content validity

Content validity refers to the reaction degree to which the content and the number of questionnaire items respond to the measured content. Content validity was determined by the content validity index (CVI). Item-level content validity index (I-CVI) ≥0.78, scale-level content validity index/universal agreement (S-CVI/UA) ≥0.80, and scale-level content validity index/average (S-CVI/Ave) ≥0.90 indicate good content validity of a questionnaire. However, the result may be influenced by the random selection of options in the questionnaire by the experts. Therefore, a random consistency correction is necessary to obtain the Kappa value (κ^*^).

##### Construct validity

The Mann–Whitney *U* test was used to assess whether the questionnaire scores were statistically significantly different between patients in the symptomatic and asymptomatic groups.

##### Criterion validity

As the gold standard for diagnosing POP, POP-Q stage is used to verify the criterion validity of the simplified Chinese version of the ICIQ-VS. Kruskal–Wallis *H* test was used to determine the relationship between the POP-Q stage and ICIQ-VS scores.

#### Sensitivity

Wilcoxon signed-rank test and paired sample *t* test were used to assess the preoperative and postoperative scores of the questionnaire. In addition, effect size (ES) and standardized response mean (SRM) were used to evaluate the sensitivity of the questionnaire. The absolute values of ES and SRM around 0.2 indicate a low sensitivity, 0.5 indicate a medium sensitivity, and ≥0.8 indicate a high sensitivity. Statistical analyses were performed using IBM SPSS 21. *p*<0.05 indicates a statistically significant difference.

## Results

In this cross-sectional study, 244 patients were enrolled (the symptomatic group, 124 patients; the asymptomatic group, 120 patients). Their ages ranged from 21 to 88 years. Moreover, 91 patients in the symptomatic group were sexually active and filled out questions about sexual matters. The median age was 47 (34.50, 62.00) years (range 21–88 years), the median parity was 2 (1.25, 3.00; range 0–7), and the median number of cesarean sections was 0 (0, 0; range 0–2). Among the 124 patients in the symptomatic group, 77 had the POP stage 2, 38 had stage 3, and 9 had stage 4. In the asymptomatic group, 95 patients were sexually active and filled out the questions about sexual matters. The median age was 40 (31.25, 53.00) years (range 21–81 years), the median number of cesarean sections was 0 (0, 0; range 0–2), and the median parity was 2 (0, 2; range 0–4). Among the 120 patients in the asymptomatic group, 53 had POP stage 0, and 67 had stage 1. The time interval between T1 (test) and T2 (retest) was 2 weeks. Among the 51 surgical patients, the age ranged from 21 to 88 years, and the median parity was 2 (2, 3; range 1–7). No patients answered the questions about sexual matters in T4.

### Reliability

#### Internal consistency

Item 9, “vagina too tight,” was primarily designed to detect a potential complication after treatment. Item 10, “Are you sexually active at present,” was a filtering item. Therefore, they were excluded from the scoring. After that, the Cronbach’s alpha coefficients of the VSS and SMS were 0.787 and 0.861 respectively. The QOL score had only one item; thus, the internal consistency could not be calculated.

#### Stability

The ICC values of VSS, SMS, and QOL score were 0.830, 0.894, and 0.948 (*p* < 0.001) respectively (Table 1). The ICC values of each item level ranged from 0.669 to 0.948 (*p* < 0.001).

**Table 1:** Test–retest reliability for the simplified Chinese version of the International Consultation on Incontinence Questionnaire-Vaginal Symptoms (ICIQ-VS)

ICIQ-VS scores	Number of patients	Initial test score	Retest score	ICC (95% CI)	*p* value
VSS	30	8.87 ± 8.21	5.90 ± 6.19	0.830(0.674–0.915)	<0.001
SMS	19	10.59 ± 12.73	9.85 ± 12.97	0.894(0.753–0.957)	<0.001
QOL	30	2.43 ± 2.79	2.17 ± 2.85	0.948(0.893–0.975)	<0.001

*ICC* intraclass correlation coefficient, *95% CI* 95% confidence interval, *VSS* vaginal symptom score, *SMS* sexual matter score, *QOL* quality of life

### Validity

#### Content validity

A total of 10 experts were invited to conduct the content validity test, and the I-CVI of the questionnaire ranged from 0.60 to 1.00. Except for the I-CVI of item 9, the I-CVI values of the other 13 items were all >0.78. The S-CVI/UA and the S-CVI/Ave of the questionnaire were 0.95 and 0.96 respectively. By calculating the random consistency probability (Pc) values and κ^*^ values of items, we concluded that the content validity evaluation of item 9 was general, and that of the other items was excellent (Table 2).

**Table 2:** Content validity for the simplified Chinese version of the International Consultation on Incontinence Questionnaire-Vaginal Symptoms

Item	I-CVI	Pc	κ*	Evaluation
1	1	0.001	1	Excellent
2	1	0.001	1	Excellent
3	1	0.001	1	Excellent
4	1	0.001	1	Excellent
5	1	0.001	1	Excellent
6	1	0.001	1	Excellent
7	1	0.001	1	Excellent
8	1	0.001	1	Excellent
9	0.60	0.205	0.497	General
10	1	0.001	1	Excellent
11	1	0.001	1	Excellent
12	1	0.001	1	Excellent
13	0.80	0.044	0.791	Excellent
14	1	0.001	1	Excellent

*I-CVI* item-level content validity index, *Pc* random consistency probability, κ* kappa value

κ* = 0.40–0.59, general; κ* = 0.60–0.74, good; κ* > 0.74, excellent

#### Construct validity

Statistical analysis was performed to test whether the questionnaire could distinguish patients in the symptomatic group from those in the asymptomatic group. The Mann–Whitney *U* test showed significantly different VSSs, SMSs, and QOL scores between the two groups (Table 3).

**Table 3:** Construct validity for the simplified Chinese version of the International Consultation on Incontinence Questionnaire-Vaginal Symptoms (ICIQ-VS)

ICIQ-VS	Group	Number of patients	Median	Range	*U* value	*p* value
VSS	Asymptomatic group	120	0	0–10		
	Symptomatic group	124	8	0–35	2,320.00	<0.001
SMS	Asymptomatic group	95	0	0–10		
	Symptomatic group	91	8	0–58	2,434.00	<0.001
QOL	Asymptomatic group	120	0	0–7		
	Symptomatic group	124	2	0–10	2,929.00	<0.001

*VSS* vaginal symptom score, *SMS* sexual matters score, *QOL* quality of life

#### Criterion validity

The Kruskal–Wallis *H* test showed a significant association between the degree of prolapse and the VSS, SMS, and QOL score (Table 4). Through pairwise comparison (Bonferroni), we found that the VSS and QOL score were statistically significantly different between patients with POP stage ≤1 and those with POP stage >1. Therefore, the questionnaire has good criterion validity and can distinguish the patients with POP stage = 0 from those with POP stage >1.

**Table 4:** Criterion validity for the simplified Chinese version of the International Consultation on Incontinence Questionnaire-Vaginal Symptoms

POP-Q	VSS		SMS		QOL	
	Median	Range	Median	Range	Median	Range
POP-Q 0	0	0–4	0	0–26	0	0–7
POP-Q 1	0	0–4	0	0–37	0	0–3
POP-Q 2	4	0–26	0	0–56	0	0–10
POP-Q 3	20	6–35	18	0–56	7	0–10
POP-Q 4	20	9–42	9.5	0–58	5	3–10
*H* value	141.89		48.82		133.29	
*p* value	<0.001		<0.001		<0.001	

*POP-Q* pelvic organ prolapse quantification, *VSS* vaginal symptom score, *SMS* sexual matter score, *QOL* quality of life

### Sensitivity

The paired sample *t* test showed that there was a significant difference in VSS before and 3 months after surgery (*t* = 19.91, *p*<0.001); the Wilcoxon signed-rank test showed a significant difference in QOL score before and 3 months after surgery (*Z* = −6.24, *p*<0.001). The ES and SRM of VSS were 2.84 and 2.79 respectively. The ES and SRM of the QOL score were 3.12 and 2.95 respectively. Therefore, the VSS and QOL score had high sensitivity (Table 5).

**Table 5:** Sensitivity analysis for the simplified Chinese version of the International Consultation on Incontinence Questionnaire-Vaginal Symptoms (*n* = 51)

Group	VSS			QOL		
	Median	Range	Mean ± SD	Median	Range	Mean ± SD
Before surgery	20	6–38	19.57 ± 6.72	7	3–10	7.00 ± 2.17
After surgery	0	0–6	0.49 ± 1.27	0	0–3	0.22 ± 0.76
*t*/*Z*	19.91			−6.24		
*p* value	<0.001^*^			< 0.001^**^		
ES	2.84			3.12		
SRM	2.79			2.95		

*VSS* vaginal symptom score, *QOL* quality of life, *ES* effect size, *SRM* standardized response mean

^*^Paired-sample *t* test

**Wilcoxon signed-rank test

*t*/*Z*, *t* test value/*Z* test value

## Discussion

The study demonstrated a successful translation of the English version of the questionnaire into the simplified Chinese version. The simplified Chinese version of the ICIQ-VS had good reliability and validity. Therefore, the questionnaire was a reliable and valid measurement tool for assessing vaginal symptoms, sexual matters, and QOL of patients with POP in China.

### Reliability of the simplified Chinese version of the ICIQ-VS

The Cronbach’s alpha coefficients of the VSS and SMS were 0.787 and 0.861 respectively, both >0.70. Therefore, the VSS and SMS had good internal consistency. The Cronbach’s alpha coefficients of the VSS and SMS of the original questionnaire and the Danish version were 0.79 and 0.84 respectively [10, 15]. The Cronbach’s alpha coefficients of the VSS and SMS of the Brazilian version were 0.79 and 0.88 respectively [11]. The Cronbach’s alpha coefficients of the VSS and SMS of the Turkish version were lower than those of other versions [16]. However, each version had a good internal consistency reliability.

To ensure the controllability of the measurement environment before and after the two investigations, 30 patients with POP were measured at an interval of 2 weeks. The ICC analysis showed that all the questionnaire items had moderate to excellent stability. In addition, the VSS, SMS, and QOL score of the questionnaire also had good stability.

### Validity of the simplified Chinese version of the ICIQ-VS

Content validity refers to the systematic evaluation of questionnaire contents and items by experts based on abundant theories and practices, mainly examining whether the questionnaire can reflect the measurement purpose and whether the content allocation is appropriate. The content validity of the original questionnaire and other versions was judged based on the missing items, whereas that of the simplified Chinese version of the ICIQ-VS was evaluated based on the CVI according to expert opinion. The I-CVI of the simplified Chinese version of the ICIQ-VS ranged from 0.60 to 1.00. Except for the I-CVI of item 9, the I-CVIs of the other 13 items were all >0.78. Although the I-CVI of item 9 was 0.60, less than 0.78, the original questionnaire development group recommended retaining the item to maintain the integrity of the questionnaire. In addition, the S-CVI/UA of the simplified Chinese version of the ICIQ-VS was 0.95, greater than 0.80, and the S-CVI/Ave was 0.96, greater than 0.90. Therefore, the simplified Chinese version of the ICIQ-VS had good content validity.

Criterion validity was based on other tools that have been validated in the field. The study revealed a significant correlation between the severity of POP and the VSS, SMS, and QOL score. In particular, the VSS and QOL score could be used to distinguish patients with POP stage ≤1 from those with POP stage >1. In the study on the Turkish population, patients with POP stage ≤1 and those with POP stage ≥2 could be distinguished by the three scores of the questionnaire [16]. In Portuguese, the VSS and QOL score could be used to distinguish patients with POP stage =0 from those with POP stage ≥3 [11].

The simplified Chinese version of the ICIQ-VS could distinguish the patients in the symptomatic group from those in the asymptomatic group. Patients in the asymptomatic group who underwent cervical cancer screening and physical examination did not have gynecological inflammation. However, other common gynecological diseases might affect the questionnaire scores. The structural validity of the questionnaire was also validated in previous studies (Danish, Turkish, Portuguese, Sinhala, and Tamil) [11, 13, 15, 16].

### Sensitivity of the simplified Chinese version of the ICIQ-VS

Sensitivity reflects the reactivity of the questionnaire. There were significant differences in the VSS and QOL score preoperatively and 3 months postoperatively. Sexual activities were routinely prohibited within 3 months of the surgery. After 3 months of the surgery, the sexually active patients still worried that premature sexual activities would affect surgery outcomes. Therefore, the patients did not fill out the questions about sexual matters. The ES and SRM of the VSS and QOL score were greater than 0.8, indicating the high sensitivity of the simplified Chinese version of the ICIQ-VS. Therefore, the simplified Chinese version of the ICIQ-VS can be used to evaluate postoperative vaginal symptoms and QOL. The significant sensitivity of VSS and QOL score was also found in Danish and Sinhalese studies; however, the SMS was less sensitive to change [13, 15]. In addition, the sensitivity of the VSS, SMS, and QOL score to change was also significant in the studies of the Turkish, Portuguese, and Tamil versions [11, 13, 10]. The sensitivity of the questionnaire was not tested in the Greek population [14].

### Limitations

Owing to multiple factors, such as human resources and financial resources, the convenience sampling method was adopted in the study, and the samples were all selected from a tertiary grade A hospital in Henan Province, which may cause a certain degree of selection bias. In addition, the menopause of patients in the symptomatic group (52 out of 124) was higher than that of patients in the asymptomatic group (36 out of 120), which may affect their sexual activities.

## Conclusion

In conclusion, the simplified Chinese version of the ICIQ-VS was successfully sinicized in this study, and the reliability and validity of the simplified Chinese version of the ICIQ-VS module were tested. The simplified Chinese version of the ICIQ-VS can provide an objective and reliable measurement tool for assessing vaginal symptoms, sexual matters, and QOL of Chinese patients with POP. In addition, the questionnaire can be used to longitudinally monitor the dynamic changes in the vaginal symptoms and QOL of patients with POP during treatment, which can provide a reference for clinical and scientific research.
